# Applications of Silver Nanoparticles in Dentistry: Advances and Technological Innovation

**DOI:** 10.3390/ijms22052485

**Published:** 2021-03-02

**Authors:** Clara Couto Fernandez, Ana Rita Sokolonski, Maísa Santos Fonseca, Danijela Stanisic, Danilo Barral Araújo, Vasco Azevedo, Ricardo Dias Portela, Ljubica Tasic

**Affiliations:** 1 Laboratory of Immunology and Molecular Biology, Health Sciences Institute, Federal University of Bahia, Salvador, BA 40140-100, Brazil; clara.fernandez@ufba.br (C.C.F.); maisa.s.fonseca@gmail.com (M.S.F.); 2 Laboratory of Oral Biochemistry, Health Sciences Institute, Federal University of Bahia, Salvador, BA 40140-100, Brazil; anton@ufba.br (A.R.S.); danilobarral81@hotmail.com (D.B.A.); 3 Laboratory of Chemical Biology, Institute of Chemistry, State University of Campinas, Campinas, SP 13083-970, Brazil; stanisicdanijela@yahoo.com (D.S.); ljubica@iqm.unicamp.br (L.T.); 4 Laboratory of Cellular and Molecular Genetics, Biological Sciences Institute, Federal University of Minas Gerais, Belo Horizonte, MG 31270-901, Brazil; vasco@icb.ufmg.br

**Keywords:** endodontics, nanotechnology, oral microbiology, periodontology, prosthetics

## Abstract

Silver nanoparticles (AgNPs) have been successfully applied in several areas due to their significant antimicrobial activity against several microorganisms. In dentistry, AgNP can be applied in disinfection, prophylaxis, and prevention of infections in the oral cavity. In this work, the use of silver nanoparticles in dentistry and associated technological innovations was analyzed. The scientific literature was searched using PubMed and Scopus databases with descriptors related to the use of silver nanoparticles in dentistry, resulting in 90 open-access articles. The search for patents was restricted to the A61K code (International Patent Classification), using the same descriptors, resulting in 206 patents. The results found were ordered by dental specialties and demonstrated the incorporation of AgNPs in different areas of dentistry. In this context, the search for patents reaffirmed the growth of this technology and the dominance of the USA pharmaceutical industry over AgNPs product development. It could be concluded that nanotechnology is a promising area in dentistry with several applications.

## 1. Introduction

The use of silver in dentistry has been documented since 1840, mainly in the prevention and treatment of dental caries [1]. Initially, it was used as silver nitrate (AgNO_3_), and then in association with fluorine (AgF). In the 2000s, silver started to be also used in restorative materials such as silver amalgam. In the 20th century, the study of nanomaterials started a new field in health sciences, then named nanotechnology. The nanometric dimension of the particles used in this new field altered the usual properties of biomaterials, showing new characteristics, processability, and capabilities [2].

Among metallic nanoparticles, silver nanoparticles (AgNP) have stood out in scientific research for presenting antimicrobial properties and biological activity against bacteria, fungi, and enveloped viruses [3,4]. The mechanism of action of AgNPs is mainly associated with the release of cationic silver and its oxidative potential [5]. Particle size and shape can also influence the mechanism of action of AgNPs, as well as their synthesis.

Therefore, silver nanoparticles emerged as a promising compound to be used in dentistry, since the incorporation of antimicrobial substances in dental biomaterials has been a strategy adopted by some researchers [6,7]. Silver nanoparticles have already proved to be effective against several multi-drug-resistant microorganisms [8,9]. However, the commercial use of silver nanoparticles (NP) in dentistry is incipient, with only three products with AgNPs in their composition being commercially available: dental adhesive (NanoCare Gold DNT™) [5,10]; Novaron AG300 (Toagosei Co Ltd., Tokyo, Japan) [11]; and sealer (GuttaFlow™ Coltène-Whaledent) [12,13].

Thus, in dentistry, the direct application of AgNP would be aimed at disinfection and the prevention against pathogenic microorganisms in the oral cavity. The main use of these particles is based on their prophylactic action. Most studies that have analyzed the use of AgNPs in dentistry did not present either further commercial and clinical applications or the chemical particularities of silver nanoparticles and their therapeutic success. Therefore, in this research, the use of silver nanoparticles in dentistry and technological innovations based on their development were analyzed. In addition, we look forward to elucidating differences between the chemical, physical, and green synthesis of silver nanoparticles, the types of nanoparticles used in dentistry, and their mechanisms of action against Gram-positive and Gram-negative bacteria and fungi.

## 2. Synthesis of Silver Nanoparticles

**Figure 1:**
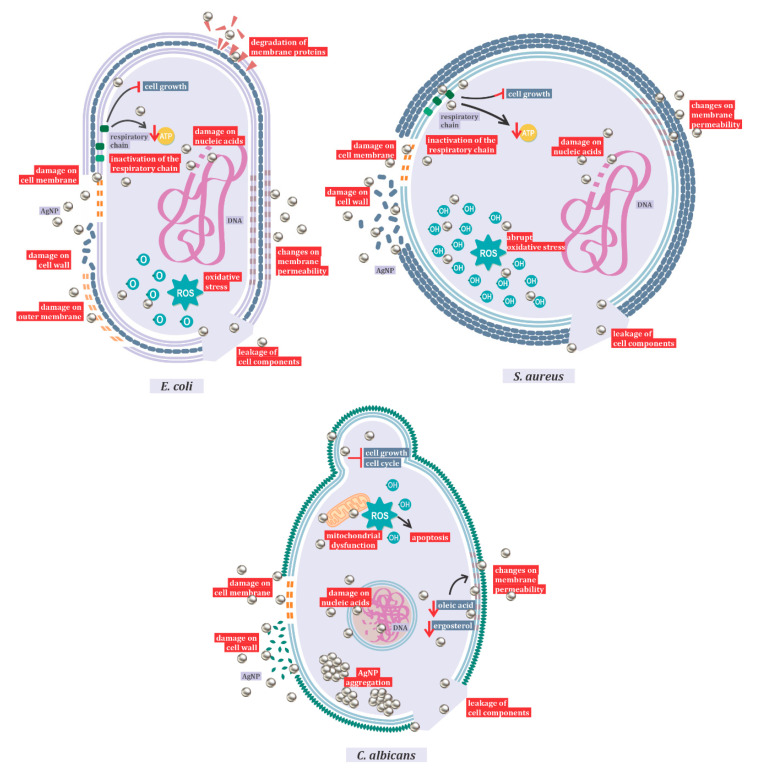
Mechanisms of action of AgNPs against *Candida albicans*, *Escherichia coli*, and *Staphylococcus aureus*.

Silver nanoparticles are synthesized using a precursor (often silver nitrate), a reducing agent that reduces silver ions from Ag^+^ to Ag^0^, and a stabilizing agent that ensures the stabilization of suspended nanoparticles and prevents nucleation and aggregation, since metallic nanoparticles have a high surface energy. Therefore, the synthesis of silver nanoparticles can be chemical, physical, or biological (Figure 1). In dentistry, the most common synthesis is the chemical route, as shown in Table 1.

The synthesis of AgNP is based on the chemical reduction of Ag^+1^ to Ag^0^. The differentiation between chemical processes is represented by the reduction agents and stabilizers used, such as sodium citrate, ascorbate, sodium borohydride (NaBH_4_), elemental hydrogen, polyol process, Tollens reagent, n,n-dimethylformamide (DMF), and poly (ethylene glycol)-block copolymers. Several protective agents (stabilizers) have been used, such as thiols, amines, acids, alcohols [93], and polymeric compounds such as chitosan [25,26,56] and polymethylmethacrylate [27,51,53,68,92]. These agents stabilize dispersive NPs during their synthesis and protect NPs that can be absorbed on, or bind onto, nanoparticle surfaces, avoiding their agglomeration and sedimentation [94].

Physical synthesis uses ultraviolet irradiation [61,65], thermal synthesis [62], and spray pyrolysis [63]. In addition, other researchers have reported unconventional synthesis by direct metal sputtering into anhydrous glycerol [95]. However, the essential approaches to physical synthesis include evaporation-condensation and laser ablation [96]. The benefit of physical approaches is the absence of solvent contamination in the preparation of thin films, the uniformity of nanoparticle distribution, high purity, and quick processing time. Small-scale production, high energy consumption, and thermal stability have been described as disadvantages [96,97]. 

However, the release of silver nanoparticles and harmful reducing agents such as sodium borohydride into the environment has become a concern. Thus, there is a search for low-cost synthesis processes and eco-friendly methods, which do not use toxic chemicals in synthesis protocols [98]. The biological synthesis emerges as a sustainable alternative and as an attempt to make the process less complicated compared to chemical and physical syntheses. It uses prokaryotic organisms, such as bacteria and eukaryotic organisms such as fungi and plants as potential reducing agents. In this method, the selection of solvents and nontoxic stabilizing agents are also taken into consideration. Our research showed that in dentistry, the most common organisms used to synthesize silver nanoparticles are plants (Table 2). Another advantage of this method is that it increases biocompatibility in living organisms, which is a desired feature for its use in human and veterinary health fields [99,100]. It is important to notice that the effectiveness of biosynthesized silver nanoparticles is related to the stabilization of the metal core with biological polymers [98,101]. 

## 3. Types of AgNPs Used in Dentistry

The biological activity of AgNPs, like other products containing silver, occurs through the gradual release of silver as a consequence of redox reactions in the presence of water [102]. In addition, the antiproliferative action against bacteria, fungi, and viruses is related to the nanoparticle size and shape, in which sizes smaller than 10 nm have higher antimicrobial activity [103]. The diversity in sizes and shapes can be explained by the different nano-ionic origins of nanoparticles [14].

In dentistry, silver nanoparticles are used in association with composites, such as Chitalac-Ag [25], AgNP-methyl polymethylmethacrylate [53,73], amorphous calcium AgNP-phosphate [52], and fluorides (Nano Silver Fluoride) [26]. It can also be used alone in the form of silver nanoparticles or silver plasma [79,87].

## 4. Mechanisms of Action of AgNPs

Silver nanoparticles are frequently associated with their antimicrobial and antioxidant activities [3]. The action of silver nanoparticles is mainly related to their nanoscale, which alters the level of silver ion release and interferes with the surface energy [5]. Nanoparticles show good antimicrobial effects due to their large surface area, providing high contact with microorganisms when compared to other antimicrobial agents [104].

The action of AgNPs against several microorganisms, including bacteria, fungi, and viruses, has already been described, showing their therapeutic potential [4]. Even multi-resistant bacteria are susceptible to AgNP, which indicates that the mechanisms that confer the resistance of these strains to commercial antibiotics have no protective activity when exposed to nanoparticles [8].

One of the most important mechanisms of action of AgNP is represented by the induction of reactive oxygen species (ROS) production, and hydroxyl radicals are the main species responsible for the oxidative damage [105]. However, it also damages the membrane and cell walls, interferes in the respiratory chain, exhausts the levels of intracellular ATP, and shatters nucleic acids [3,5]. This mechanism of action varies with nanoparticle size and shape, and with the different target species. In this review, the mechanism of antibacterial action against Gram-positive and Gram-negative bacteria and the antifungal mechanism against *Candida albicans* (Figure 1) were highlighted. In Gram-negative bacteria, with *Escherichia coli* as a representative species, studies have shown action primarily on the outer membrane, resulting in the leakage of cell components.

After entering the cell, it has also been shown that AgNPs inactivate the respiratory chain dehydrogenases, inhibiting cell growth and respiration. In addition, these nanoparticles can act on phospholipids and membrane proteins, causing a breakdown in the plasma membrane and changes in its permeability [106]. The main responsible for the oxidation of lipids in *E. coli* is reactive oxygen [105]. Electron microscopy analyses indicated the fragmentation of *E. coli* after treatment with silver nanoparticles [106]. Gram-negative bacteria exhibited no resistance to the antimicrobial action of silver [2].

The difference between the action of silver nanoparticles on Gram-positive and Gram-negative bacteria is related to the structure of the peptidoglycan cell wall. When comparing inhibition between *Escherichia coli* and *Staphylococcus aureus*, the latter being considered as a model microorganism for Gram-positive bacteria studies, it was observed that Gram-negative bacteria are more easily inhibited than Gram-positive ones [107]. Gram-positive bacteria also show changes in membrane permeability and protein composition in the respiratory chain, and the formation of ROS [107]. Oxidative stress in Gram-positive bacteria is more abrupt than in Gram-negative ones. As in Gram-negative bacteria, high ROS concentrations lead to protein degradation by activation of the proteolytic pathway and lipid oxidation. However, in *S. aureus*, the hydroxyl radical is responsible for lipid oxidation. As in Gram-negative microorganisms, there are also changes in membrane potential, as well as DNA degradation in Gram-positive bacteria [105].

When the mechanism of action of silver nanoparticles in bacteria and fungi is compared, the aggregation of nanoparticles only occurs in eukaryotic cells, resulting in larger particles [98]. In *Candida* species, it has been shown that the toxic action of AgNP is related both to the ROS-mediated pathway, inducing dysfunctional mitochondrial apoptosis, and to the ROS-independent pathway, culminating in the same cell death outcome [108]. Similar to the antibacterial action, in *Candida* species, AgNP acts by interfering with the membrane potential, in its integrity and fluidity, in its growth, and in the cell cycle [108,109]. In addition, the synthesis method influences the action of silver nanoparticles, with biosynthesis showing better results [110].

A brief description of the activity of silver nanoparticles in Gram-positive, Gram-negative bacteria and fungi is shown in the Supplementary Tables S1–S3, respectively. These tables also describe the type and size of the particles, as well as the synthesis methods that were used in each study. 

## 5. Silver Nanoparticles and Dentistry

**Figure 2:**
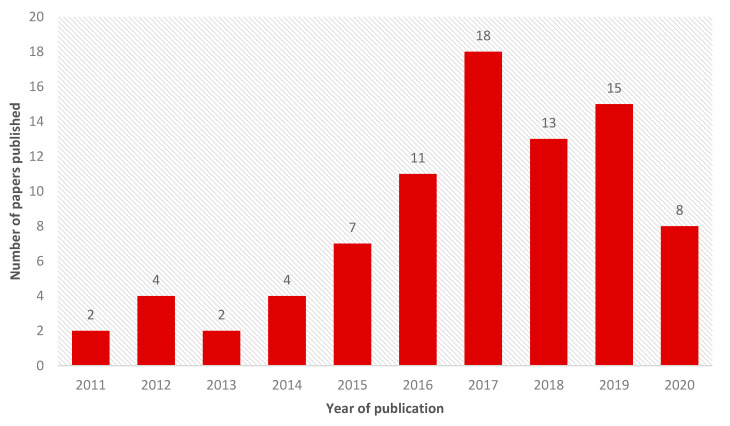
Number of scientific reports on the use of silver nanoparticles in dentistry by year, focusing on the last decade (2010–2020).

The use of silver in dentistry dates from the 19th century and has different applications, mainly due to the antimicrobial potential of silver ions [1]. However, in the 21st century, the advent of nanotechnology brought a new perspective on the use of silver in dentistry through silver nanoparticles, which have antimicrobial action mainly due to the gradual release of silver ions [49,50,53,65]. The use of nanoparticles in dentistry over the years is evidenced by the number of articles published in the last ten years (Figure 2). 

In dentistry, studies have indicated the use of silver nanoparticles in different specialties: oral microbiology, preventive dentistry, prosthodontics, orthodontics, endodontics, and periodontics. In addition, some studies have investigated the potential of using silver nanoparticles by testing their antimicrobial effects against the most common oral pathogens. Considering the use of AgNP in the different dentistry specialties and subsequent fields, the predominant areas are dental prosthesis (25.6%) and oral microbiology (19.5%) (Table 3).

### 5.1. Oral Microbiology

The oral cavity is a microbiome inhabited by more than 700 bacterial species [111] of fungi, viruses, and protozoa [112]. This microbiome is a crucial factor in understanding the etiology of many oral and systemic diseases [112,113,114], being in many cases a determinant of health and disease statuses. For the maintenance of the oral and systemic health, the balance of the oral microbiota is vital [111]. Studies have revealed that the most abundant microorganisms in the healthy oral microbiome belong to the Firmicutes, Proteobacteria, Fusobacteria, and Actinobacteria phyla. The genus Streptococcus is the most prevalent, followed by Prevotella, Veillonella, Neisseria, and Haemophilus [115]. 

Studies have also revealed that AgNP exerts antibacterial activity against Streptococcus mutans [3,42,49,60,69], *Staphylococcus aureus* [3,54,58,69,74,93], *Streptococcus sobrinus*, *Lactobacillus acidophilus*, *Lactobacillus casei* [42], *Streptococcus sanguinis* [49], *Enterococcus faecalis* [69,93], and Actinomyces actinomycetemcomitans [49]. AgNPs also prevent the formation of *E. faecalis*, *S. aureus*, *Streptococcus gordonii*, *Streptococcus mitis*, and *Streptococcus mutans* biofilms [30]. However, it is essential to understand that these works analyzed biofilm formation in monocultures. Dental caries in the oral cavity have complex multispecies biofilms, and thus it is not possible to extrapolate in vitro data to the clinical use of AgNP [30]. Nanoparticles already exhibited strong antimicrobial activity against Gram-positive and Gram-negative bacteria in planktonic, agar-grown, or biofilm cultures. Despite their thick, protective outer peptidoglycan layer, Gram-positive bacteria are very vulnerable to the antimicrobial action of silver [30]. Gram-negative bacteria such as Escherichia coli and Pseudomonas aeruginosa exhibit no resistance to the antimicrobial action of silver [30,54,58,74,93].

The antimicrobial efficacy of nanosilver is inversely proportional to the size of silver nanoparticles [15]. Silver nanoparticles with smaller diameter present better biofilm inhibition results than larger particles [31] and are effective against S. mutans [33] and Streptococcus orallis biofilms [88]. However, larger AgNPs were also demonstrated to have significant antimicrobial activities against a wide variety of dental plaque microorganisms, showing acceptable bacterial growth inhibition even at lower concentrations [31]. Biologically synthesized AgNP (using neem, onion, and tomato in the synthesis process) with sizes considered large (26.2 to 33.3 nm) showed antimicrobial activity against *S. aureus*, possibly due to the high concentration of flavonoids and terpenoids [75]. 

AgNPs were more effective than chlorhexidine against *Enterococcus faecalis*, *Klebsiella pneumoniae*, *C. albicans* [72], and *S. mutans* [15]. Other studies have reported that pure chlorhexidine has higher antimicrobial activity against *E. faecalis* and *C. albicans* [17,31], and a positive synergistic effect was present when AgNP was associated with chlorhexidine [72] biocomposites such as calcium glyceride phosphate [50] or antibiotics [76].

### 5.2. Pediatric Dentistry

Pediatric Dentistry is an age-defined specialty that provides both primary and comprehensive preventive and therapeutic oral health care for infants and children through adolescence, including those with special health care needs [116]. 

Glass ionomer cement (GIC) has many uses in pediatric dentistry and is known by its fluoride release and storage capacity. This release makes this cement an anti-caries agent, as fluoride inhibits bacterial enzyme enolase. However, this material needs to be replenished with fluoride from time to time to maintain its anti-caries effect. In this context, the impregnation of GIC with longer-lasting antimicrobial agents would make this cement more effective in combating oral diseases. The association between GIC and AgNP generated a biomaterial with antimicrobial action against Gram-positive and Gram-negative bacteria [5,65]. The authors believe that the antimicrobial action occurs through the release of silver ions, which causes an oxidative dissolution in the cement matrix, inhibiting dental caries and preventing the development of oral biofilms. The union of these materials presents mechanical parameters like commercial GIC [65]. In disagreement, another study claims that this union decreases GIC hardness and is cytotoxic [81]. However, when 12 nm AgNPs were tested associated with GIC, they did not present cytotoxicity to odontoblastic lineage cells [40]. In addition, AgNP immobilization in Halloysite Nanotubes (HNT/Ag) and its incorporation on novel experimental dental resin composite inhibits the growth of *S. mutans* with no associated cytotoxicity [57]. Researchers have incorporated AgNP into a resin matrix based on bisphenol A-glycidyl methacrylate/triethylene glycol dimethacrylate (BISGMA/TEGDMA), which is used in restorations of deciduous and permanent dentitions through chitosan polymers. They found antimicrobial activity against *S. mitis*, pointing out that the coating of restorative materials by this polymer decreases antimicrobial activity [25]. However, incomplete nanocomposite polymerization (resin + AgNP), along with an increase in the release of unbound monomers, has been demonstrated [117]. The literature does not clearly state whether AgNPs can be used with polymer resins in restorative dentistry [118,119]. 

The union of AgNP with composite resins did not decrease the marginal infiltration of the material [86]. These nanocomposites have their final mechanical properties influenced by the type of polymerization [21]. The use of photopolymerization for the formation of resins associated with silver nanoparticles did not improve the mechanical properties when compared to commercial resins [64].

Dentin adhesives associated with AgNPs increased surface wetting and cohesive failures [43]. When self-etching adhesives and AgNP were tested against *S. mutans,* antimicrobial activity was observed, without compromising the conversion of adhesive into the resin [22], and AgNP incorporation made the antibacterial activity more durable. It can be applied for immediate antibacterial needs [63]. 

Two-step adhesive systems associated with AgNP showed higher shear strength results than self-etchers/AgNP [55]. AgNP powder showed better results than the alcoholic AgNP solution both in antimicrobial activity and in the degree of conversion of the self-etching adhesive [22]. AgNP incorporation in disinfectants generated commercial products (Nanocare Gold) with biocompatibility and no cytotoxicity to stem cells from dental pulp [5].

#### Preventive Dentistry

Dental caries is a dysbiotic disease of polymicrobial etiology caused by the imbalance between demineralization and remineralization [120,121]. Dentistry seeks to combat caries by controlling the microbiota and stimulating the remineralization of incipient lesions on the enamel surface. This treatment is widely used in primary teeth.

Silver ions can infiltrate carious lesions and precipitate, resulting in enamel hardening. For the remineralization of incipient lesions, dental surgeons use sodium fluoride varnish in the clinical routine. However, when 5% of nanosilver is added to the sodium fluoride varnish, there is a 77% inhibition of the progression of caries lesions in residual teeth, without the presence of a metallic taste or painful ulcerations [39]. Silver Nanofluoride (NSF) is easy to administer, can be applied only once a year, has a reasonable cost-benefit ratio, and can be used to replace varnish with sodium fluorine [14], or the traditional silver compound, Silver Diamine Fluoride (SDF). NSF is a bacteriostatic agent, as it inhibits the growth of *S. mutans* biofilm [26], and is also capable of paralyzing caries activity, so it can be used as a preventive treatment without staining children’s teeth [26,56]. In artificial enamel caries, AgNPs associated with graphene oxide (rGO/Ag) [47] composites and AgNPs associated with a 650 nm Laser [80] decreased the demineralization of artificial enamel caries in a biofilm *S. mutans* model.

### 5.3. Orthodontics

The presence of fixed orthodontic appliances on teeth surfaces hinders the cleaning process, leading to dental biofilm accumulation [122]. After the application of orthodontic appliances, there are increases in the amounts of *Streptococcus mutans* and *Lactobacilli* spp. in saliva, dentition, and plaque formation [123]. Incipient caries lesions, known as white spots, are the most common complications in patients using fixed orthodontic appliances, especially when there is poor oral hygiene [51]. Elastomeric modules, brackets, orthodontic wire, and titanium micro-implants were treated with AgNP with the objective of avoiding this condition [31,49,71].

AgNP treatment showed a decreased demineralization in patients undergoing orthodontic treatment and an antibacterial activity against *E. coli*, *L. casei*, *S. aureus*, *S. mutans* [71]. AgNP showed non-stick biological properties in wires [31], and brackets against *S. mutans* [31,54,90]. It is essential to highlight that only one study tested different sizes of silver nanoparticles, showing better results with smaller particles [31].

The decrease in the incidence of dental caries on smooth surfaces after AgNP impregnation revealed that the antibacterial activity of silver nanoparticles has characteristics of contact inhibition and not only with ion release [90]. Nanoparticles were also incorporated into acrylic resins, base plates of orthodontic appliances, and inhibited biofilm formation and planktonic growth [42]. Titanium micro-implants treated with 21% AgNP and biopolymer demonstrated antimicrobial activity [49], as well as AgNP and GIC composites used in orthodontic cementation, which were also able to reduce the biofilm’s metabolic activity and the bacterial acid production [51]. 

### 5.4. Endodontics

The crucial cause of apical periodontitis is inflamed or necrotic pulp, which is a consequence of colonization by microorganisms and can even lead to bone infection [109]. In infected root canals, despite its polymicrobial etiology, *Enterococcus faecalis*, a facultative anaerobic Gram-positive bacterium, is often present, causing persistent and difficult-to-treat infections. The association of AgNP with composites disrupts E. faecalis biofilm through the release of silver ions [29]. AgNPs also demonstrated their antimicrobial effect when used as final endodontic irrigators, with the effect similar to the treatment with 2.5% sodium hypochlorite [34].

Calcium-based cement and mineral trioxide aggregate (MTA) associated with AgNP presented antimicrobial activity against *Escherichia coli*, *Actinomyces* spp., *Streptococcus mutans*, *E. faecalis*, and *C. albicans* isolates. Silver particles can decrease the attachment of microorganisms to the tooth surface and increase the antibacterial properties of endodontic sealers [23]. These particles also increased the MTA radiopacity [91].

### 5.5. Periodontics

The action of silver nanoparticles is not restricted to microorganisms that cause dental caries but can also be active for other cells and tissues of the oral cavity. Thus, some studies have demonstrated the action of AgNPs on human gingival fibroblasts [20,36] and human oral keratinocytes [89].

The main issue verified when analyzing the use of AgNP in dentistry refers to determining the ideal concentration toxic to microorganisms and not cytotoxic to the patient’s cells, ensuring that no damage to healthy tissues occurs. An important finding demonstrated that 2 nm AgNPs at 1.5 ug/mL concentration have no cytotoxic activity. At the same time, the association of AgNPs with fluorine or sodium fluoride [36] increases oxidative stress in gingival fibroblasts, leading to tissue inflammation that results in apoptosis and compromises cell viability [20].

The strategy of capping silver nanoparticles can improve biocompatibility by creating surface functionalization [36]. 10 nm nanoparticles capped with lipoic acid or polyethylene glycol decreased the cytotoxic effects against human gingival fibroblasts at nontoxic concentrations (<50 μg/mL) and showed marked antimicrobial potential [36], inhibiting methicillin-resistant *S. epidermidis* and *S. mutans* strain biofilms. In human oral keratinocytes, it was found that high AgNP concentrations cause the activation of NLRP3 inflammasomes, a reduction in the number of acidic organelles, and cathepsin B expression, showing that cytotoxicity is related to lysosomal damage and to the inflammatory processes [89].

### 5.6. Prosthodontics

This specialty includes diagnosis, treatment planning, rehabilitation and maintenance of the oral function, comfort, appearance, and health in clinical conditions associated with missing or deficient teeth and/or oral and maxillofacial tissues using biocompatible substitutes [116]. In this review, this specialty was divided into two fields: dental implantology and dental prosthesis.

#### 5.6.1. Dental Implantology

One of the most common causes of implant failure is peri-implantitis, which is caused by the formation of bacterial biofilm on the surfaces of dental implants. The modification of surface nanotopography has been suggested to affect the bacterial adherence to implants [78].

Titanium discs treated with AgNP-based composites decreased the biofilm adhesion and lactate production by microorganisms, despite presenting some cracks [78]. When titanium discs are coated with hydroxyapatite and AgNP, they show activity against *E. coli* [66], and the amount of AgNP is defined by the time of electrochemical reduction [66]. Using the Tollens reagent method (an eco-friendlier chemical synthesis), the 0.05 ppm concentration was sufficient for a good antimicrobial activity against common pathogens in the oral mucosa. However, the prolonged AgNP deposition negatively interfered in the surface properties, increasing the roughness and hydrophobicity, and oral bacteria are more likely to adhere under these conditions. The 0.1 ppm concentration was toxic to human osteoblasts [46]. When applying an AgNP suspension on the surface of implants with hexagonal connections, *C. albicans* contamination was reduced when torques smaller than the manufacturer’s recommendation were used [24]. 

Ag-Fe_3_O_4_ nanocomposite associated with poly lactic-co-glycolic acid (PLGA) coating dental implants under an extracorporeal magnetic field used to fix NP on the implant surface weakened *S. mutans* adhesion. In addition, it did not induce ROS production by the immune system, and the implant microenvironment presented a stimulated osteoblastic proliferation [67]. A lasting antibacterial effect against *E. coli* was achieved when hydrogen titanate nanotubes were treated with AgNP, because it offered a long-term Ag^+^ release [44]. When titanium is treated with two different silver and polyoxoxamine (PDA) concentrations, it exhibits antibacterial activity against *S. mutans* and *P. gingivalis* [61]. AgNPs can also be directly formed on the titanium plate, with antibacterial effect against *S. mutans,* without cytotoxicity to human dental pulp stem cells [48].

Alternatively, implants treated with Silver Plasma demonstrate better osteointegration results than those treated with acid [79]. Another study suggested that by using proper Silver plasma conditions, titanium implants with hierarchical micro/nanostructures can have antibacterial effects against both Gram-positive *S. aureus* and Gram-negative *Fusobacterium nucleatum* [87]. The guided tissue regeneration of membrane impregnated with silver nanoparticles increased the tensile strength and minimized the fiber diameter of the biomaterial [77]. The association of AgNPs with NRL (Natural Rubber Membrane) decreased cytotoxicity and presented 98% cell viability [59].

#### 5.6.2. Dental Prosthesis

Dentistry uses a series of compounds based on BISGMA/TEDGMA, PMMA, silicones, alginates, tissue conditioners, and porcelain to make molds and prosthetic devices. The addition of silver nanoparticles to silicones promoted an antibacterial effect proportional to the AgNP concentration [19], and their addition to alginates did not alter the mechanical properties of the impression material [83], but decreased the setting time and increased the solubilization of Portland cement [37].

Clinical evidence shows that prosthetic devices based on PMMA resins suffer from *C. albicans* infections [124,125,126], which affects their useful life. Several protocols for periodic chemical cleaning of prostheses have been proposed to eradicate such infections. However, these solutions are not definitive, and treatment repetition causes damage to the prosthesis surface, compromising the longevity of implants and prosthetic devices [127,128,129].

Based on these observations, structural modifications of PMMA matrices at nanoscale or through combination with composites could be a strategy to improve their performance. As an example, the addition of 1% silver graphene improved the mechanical properties of PMMA [68], including the increase of their viscoelastic properties [84]. The addition of AgNPs to PMMA decreased surface roughness and reduced *C. albicans* viability due to the reduced ability of the fungus to adhere and colonize dental prostheses [35,53,70,73]. In disagreement, other researchers have reported that AgNP did not inhibit *C. albicans* growth [32], but when associated with quaternary ammonium dimethacrylate (QADM), they presented antibiofilm activity [52]. The association of titanium dioxide and AgNPs showed antimicrobial properties but did not improve the mechanical properties of the material [92]. However, other authors have reported the ability of AgNPs to reduce the flexural strength of composites [18].

When AgNPs were incorporated into ethylene-vinyl acetate copolymer masterbatch, they promoted a bacteriostatic effect by inhibiting the growth of *E. coli*, *Streptococcus sobrinus*, and *Porphyromonas gingivalis* without damaging the mouthguard during physical exercise [62]. This antibacterial effect of AgNP on acrylic resins has been previously reported [27]. When nanoparticles were mixed with PMMA, amorphous calcium phosphate, 2-methacryloyloxyethyl phosphorylcholine (MPC), and dimethylaminohexadecyl methacrylate in a multifunctional biogenic composite, they reduced root dentin demineralization [38].

AgNPs incorporated into porcelains increased the fatigue parameter of this material and consequently increased its useful life [85] and increased fracture resistance [28]. The addition of 1% silver nanoparticles from 100 to 120 nm in tissue conditioners promotes their antibacterial effect, while an antifungal effect is only obtained with 2% AgNP concentration [45].

On the other hand, the addition of AgNPs caused color changes in prosthetic devices [16,53,70,84]. Color change was attributed to the plasmatic effect of AgNP through electronic propagation as an electromagnetic wave in the visible light spectrum [16,29]. In addition, researchers have already reported that AgNPs have no genotoxicity [73] or cytotoxicity [27,35,37,82]. In contrast, another study has already reported mild cytotoxicity in rat fibroblasts [92].

## 6. Biodistribution, Elimination, and Toxicity of AgNPs

One of the major aspects to be elucidated on the development of different therapeutic protocols involving silver nanoparticles is its pharmacokinetics. The knowledge of the mechanisms of absorption, biodistribution, metabolism, and elimination of silver nanoparticles is fundamental, since it is directly related to their toxicity, as well as their concentration on the blood, other tissues, and organs.

The oral administration of silver and AgNPs showed that the nanoparticles were less absorbed, reaching a higher fecal excretion and lower levels in organs [130]. Indeed, when citrate-coated nanoparticles were orally administered to rats, it was found that AgNP blood levels were very low, and high amounts of nanoparticles were found in the feces [131]. The most common AgNP uptake mechanism into intestinal epithelial cells is endocytosis [132]. Size can influence the absorption of AgNPs, and nanoparticles larger than 300 nm were shown not to be absorbed [133].

It was already demonstrated in experimental models using mice and rats that AgNPs accumulate preferentially at the liver and spleen, but they can also be detected in other organs, such as the kidneys, heart, and lungs [134,135]. In these organs and in the blood, the main cellular type responsible for their clearance are the professional macrophages of the mononuclear phagocyte system, and the nanoparticles’ clearance is directly dependent on the global status of the immune system [136]. Nanoparticles are described as being more resistant to elimination through metabolism in the liver and excretion through urine [137]. Indeed, AgNPs stabilized with polyvinylpyrrolidone were preferentially excreted by rats through their feces, and the excretion via urine was very low [134].

The toxicity of silver nanoparticles and their associated risks have been a source of concern for researchers. The risks of using nanotechnology include not only risks to the health of patients but also risks associated with the disposal of this material into the environment.

Some researchers argue that nanoparticles release silver cations in a controlled manner, so that the antimicrobial activity is carried out in small doses of silver ions released into the medium, not constituting a toxic threat to the patient [15]. However, another study has shown that the AgNP concentration is indeed a concern, showing that there is a threshold between concentrations considered toxic to microorganisms and those considered toxic to the patient’s cells [36,89]. In addition to using the correct concentration, the use of capping with organic molecules, such as chitosan, or the use of biosynthesized nanoparticles, increases biocompatibility and decreases cytotoxicity.

It was already noted that the intragastric administration of AgNPs did not result in rats’ lethality or pronounced toxic effects, and did not influence the hematological and biochemical parameters [138]. Indeed, ionic silver, which has been widely used in dentistry, is likely more toxic than nanoparticulated silver [139]. A study conducted with human volunteers [140] showed that a fourteen-day oral dosing of silver nanoparticles did not cause evident metabolic or hematologic changes, nor changes in urinalysis parameters, overall physical state, or imaging morphology. But it is noteworthy that the toxicity of 5 nm nanoparticles was described on human endothelial and bronchial epithelial cells [141], and this study used microarray analysis to show that AgNP-treated cells presented significant variations in cell death-, apoptosis-, and cell survival-related gene expression; however, 100 nm silver AgNPs did not induce cell death even at high concentrations, showing that AgNP toxicity is highly affected by the size of the nanoparticle.

One of the major concerns regarding the utilization of silver nanoparticles is their use in pregnant women and animals. It was already demonstrated that when pregnant mice were exposed to 18–20 nm AgNP, silver-containing nanoparticles could be detected in the placenta and in the head of the fetus. In the fetus, silver was detected in the ionic form or as nanoparticles with a size less than 13 nm [142], and this situation points to precautions with respect to acute exposure to nanoparticles during pregnancy. This accumulation of silver in the central nervous system has already been shown to induce long-term memory impairments in a mice model [143]. In a similar way, phytoreduced silver nanoparticles with polyphenols from *Viburnum opulus* fruit extract presented testicular toxic effects in offspring during the embryological development of the murine gonad [144]. Therefore, more studies are necessary to evaluate if nanoparticles can be safely administered to pregnant women.

Some studies also associated silver nanoparticles with commercial antibiotics, especially for the inhibition of multi-drug resistant strains [118,119]. Silver nanoparticles containing antibiotics and biologically synthesized using chitosan presented low toxicity with minor hepatotoxicity at higher doses, as shown by a biochemical and histopathological analysis [145]. However, it was described that the acquisition of *E. coli* and *P. aeruginosa* resistance after repeated exposures to low AgNP concentrations occurred by the phenotypic increase in the production of flagellin [9].

Regarding risks to the environment, the biosynthesis of metallic nanoparticles is often associated with lower toxicities to ecosystems, mainly because it does not use harmful chemicals [71,103]. It was already observed that, when studying plants and animals that can be considered as bioindicators of environmental contamination, the acute toxicity of AgNPs was rarely observed in algae, crustaceans, and fish, while it was significantly detected in cnidarians [146]. However, it was demonstrated that the intake of AgNP by zebrafish led to a significant accumulation of nanoparticles in the liver, intestine, and gill [147].

## 7. Technological Innovation

With the advent of multidrug-resistant microorganisms and the superinfections caused by single pathogens or their associations, it is necessary to develop technological innovations that can act to solve these problems. In this sense, the search for patents can give an overview of the scientific research and the commercial scenario of the use of silver nanoparticles in the medical sciences.

**Figure 3:**
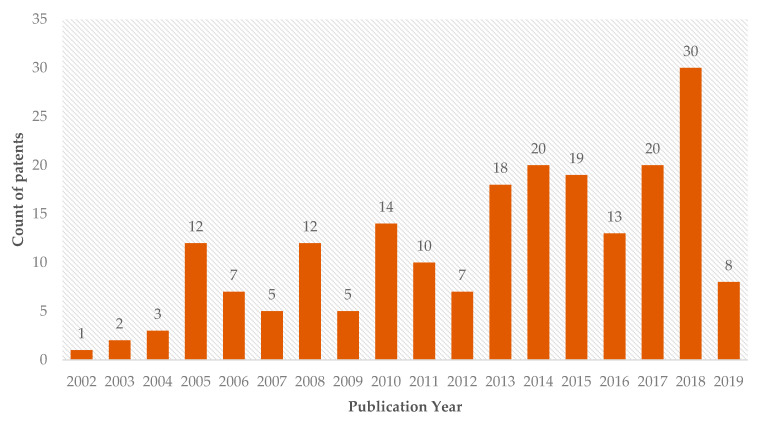
Patents issued on the use of silver nanoparticles in dentistry by year of publication.

The search for patents that use silver nanoparticles in health applications resulted in 206 patents, with the first one applied in 2002, showing that this is a recent technology under constant development (Figure 3), which corroborates the literature that addresses the use of nanotechnology in health areas from the end of the 20th century [2].

**Figure 4:**
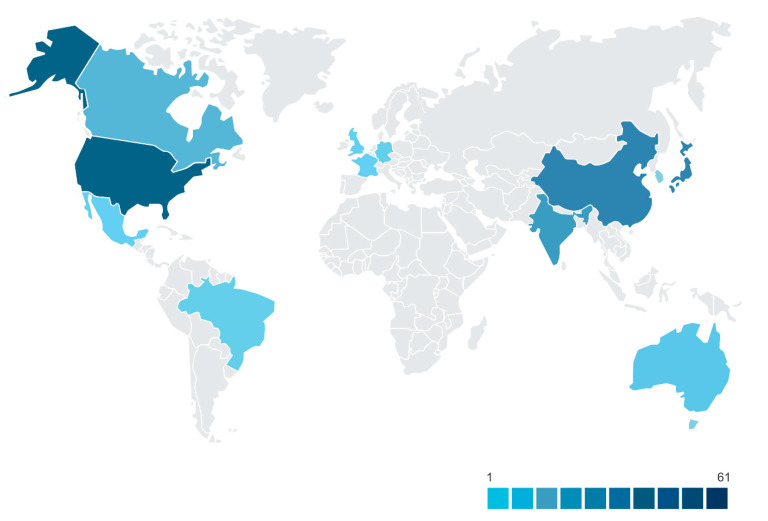
Patents issued on the use of silver nanoparticles in dentistry by country of publication.

The USA is still the country with the highest protection, showing a trend in the market and the pharmaceutical industry to use this technology. Protection also occurs in China, India, Canada, and Brazil (Figure 4).

**Figure 5:**
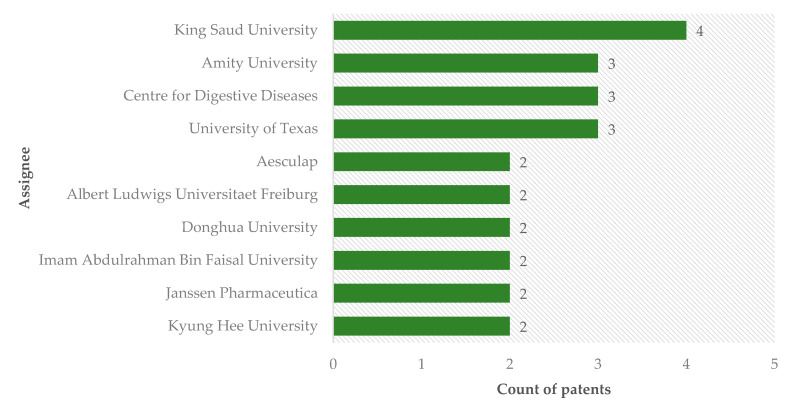
Patents issued on the use of silver nanoparticles in dentistry by depositors.

Patent analysis also shows that universities are the main depositors of this technology, becoming the cradle of nanotechnology research (Figure 5). Among the ten main assignees, seven are universities, demonstrating that this technology is practically being developed within educational institutions. In addition to universities, some companies are also depositors. Aesculap Inc., a multinational company located in the United States, working in the field of products and services for surgical procedures, and Janssen Pharmaceutical, located in Belgium, linked to Johnson & Johnson’s, which is dedicated to pharmacological research, are some examples of companies dedicated to this issue.

**Figure 6:**
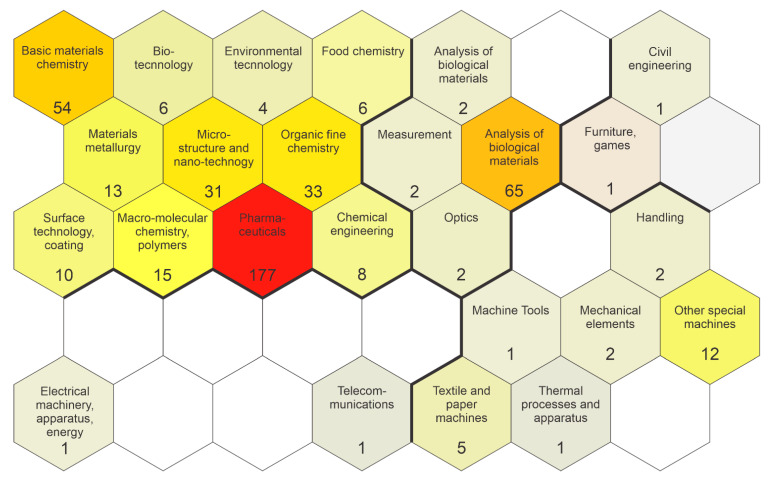
Patents issued on the use of silver nanoparticles in dentistry by the technology domain of patents.

When analyzing the technological domains of patents, it was observed that most of them are related to the pharmaceutical domain, among other technological fields (Figure 6), mainly due to the antimicrobial activity of silver nanoparticles. In dentistry, the range of technological domains is even more evident since AgNPs are commonly associated with biomaterials, such as the invention of the silver-PMMA nanocomposite film based on *Aristolochia bracteolate* with inhibition activity on *E. coli* and *Bacillus cereus* growth in aqueous solutions [148].

Another invention concerns a method of depositing silver nanoparticles on the dental implant surface, aiming to add antimicrobial properties to this material [149]. This is an example of the pharmaceutical technological domain and of surface and coating technology. Some patents also include preventive dentistry, such as oral and throat care agents with antimicrobial, antifungal, and local anesthetic action that can be daily used. In addition, they have organoleptic properties, essential for dental treatments, strengthening gums and reducing gingival bleeding [150].

The analysis of patent deposition over the years, as well as the increase in scientific research using silver nanoparticles, shows that nanotechnology has been consolidated as a therapeutic strategy and an antimicrobial alternative in dentistry. However, the development of this technology is still restricted to academia, resulting in only two commercially available products.

## 8. Future Outlooks

In dentistry, only three commercial products with AgNPs in their composition were patented until now: NanoCare Gold DNT™ (Dental Nanotechnology Ltd., Katowice, Poland) [5,10]; Novaron AG300 (Toagosei Co Ltd., Tokyo, Japan) [11]; and GuttaFlow™ (Coltène-Whaledent, Altstätten, Switzerland) [12,13]. However, there is a worldwide growth in publications and technological development on AgNP in the health area, indicating the increase in research on this technology, which has already proven the antimicrobial activity of AgNPs alone, in nanocomposites, or associated to biomaterials. Thus, AgNPs emerge as an antimicrobial agent for use in the control of pathogenic bacteria, caries activity, tissue inflammation, and bone loss, when at concentrations presenting low cytotoxicity to the patient’s cells.

## 9. Data Search

The scientific literature was searched using PubMed and Scopus databases with descriptors related to the use of silver nanoparticles in dentistry, focusing on scientific reports published in the January 2010–November 2020 period. The search was performed using “TITLE-ABS (((silver AND nanoparticles) OR agnp OR (nano AND silver)) AND (dental OR dentistry)) AND (LIMIT-TO (SRCTYPE, “j”)) AND (LIMIT-TO (DOCTYPE, “ar”))”, resulting in 95 open-access articles. The following exclusion criteria were used: (1) papers that did not report use in dentistry and did not evaluate antimicrobial activity against oral pathogens, (2) articles without application in dentistry, and (3) studies that did not evaluate silver nanoparticles. The search resulted in 82 papers in this review. Studies that used silver nanoparticles solutions or silver to construct nano compounds were included.

An Orbit Intelligence search was performed using the same descriptors and Boolean operators to accomplish innovation data. However, as silver nanoparticles are widely used in fabrics with anti-deodorant action, which is not the aim of this study, this information was added to the search. The search for patents was restricted to the A61K code (International Patent Classification), which corresponds to Preparations for Medical, Dental or Hygienic Purposes. Thus, the search mechanism was (nanosilver OR silver nanoparticle* OR AgNP) AND (antimicrobial* OR anti-inflammatory OR antibiotic*) NOT (cloth* OR textile*) AND A61K. These searches found 206 patents.

## Figures and Tables

**Table 1:** Synthesis methods of silver nanoparticles used in dentistry.

Synthesis Method	References	Total	%
Commercial synthesis	[5,14,15,16,17,18,19,20,21,22,23,24]	14.6	
Chemical synthesis	[25,26,27,28,29,30,31,32,33,34,35,36,37,38,39,40] [41,42,43,44,45,46,47,48,49,50,51,52,53,54,55,56,57] [58,59,60]	43.9	
Physical synthesis	[61,62,63,64]	4.9	
Physicochemical synthesis	[65,66,67,68]	4.9	
Biosynthesis	[3,69,70,71,72,73,74,75,76,77]	12.3	
Uninformed	[78,79,80,81,82,83,84,85,86,87,88,89,90,91,92,93]	19.5	

**Table 2:** Species used for the biosynthesis of silver nanoparticles.

Reference	Kingdom	Species
[69]	Algae	*Spirulina platensis*
[70]	Fungae	*Fusarium oxysporum*
[71]	Plantae	*Heterotheca Inuloides*
[72]	Plantae	*Cassia roxburghii*
[73]	Plantae	*Geranium maculatum*
[75]	Plantae	*Allium cepa*, *Azadirachta indica*, *Solanum lycopersicum*
[76]	Plantae	*Salix alba*
[77]	Plantae	*Aloe vera*
[3]	Plantae	*Triticum aestivum*
[74]	Viridae	*M13 phage*

**Table 3:** Dental specialties and studies on silver nanoparticles.

Dental Specialties	References	Total	%
Oral microbiology	[3,15,17,30,31,33,50,58,60,69,72,74,75,76,88,93]	19.5	
Pediatric dentistry	[5,21,22,25,40,44,55,57,64,65,81,86]	15.9	
Preventive dentistry	[14,26,39,47,56,80]	7.3	
Prosthodontics			
Dental implantology	[24,44,46,48,59,61,66,67,77,78,79,87]	14.6	
Dental prosthesis	[16,18,19,27,28,32,35,37,38,45,62,70,73,83,84,85,92] [52,53,68]	25.6	
Orthodontics	[41,42,49,51,54,71,90]	8.5	
Endodontics	[23,29,34,91]	4.9	
Periodontics	[20,36,89]	3.7	

## Supplementary Materials

The following are available online at https://www.mdpi.com/1422-0067/22/5/2485/s1, Table S1: Activity of silver nanoparticles on Gram-positive bacteria. Table S2: Activity of silver nanoparticles on Gram-negative bacteria. Table S3: Activity of silver nanoparticles on pathogenic fungi.
